# Ectopic Gestation

**Published:** 1896-09-19

**Authors:** W. H. C. Newnham

**Affiliations:** Assistant Physician Accoucheur, Bristol General Hospital


### ECTOPIC GESTATION.

By W. H. C. Newnham, M.A., M.D.Cantab., Assistant Physician Accoucheur, Bristol General Hospital.

This phrase is by far the best which can be applied to this curious and most interesting displacement, for it gives a convenient and complete definition without expressing any theoretical explanation of the condition. All ectopic gestations are probably tubular pregnancies, but it is hardly fair to adopt that title, as there are some who still cling to the belief of the occurrence of the ovarian kind. My view is that the uterus alone is the seat of normal conception, that as soon as the ovum is affected by the spermatozoa it adheres to the mucous surface of the uterus. The function of the ciliated lining of the Fallopian tubes is to prevent the spermatozoa entering them, and to facilitate the progress of the ovum towards the uterus. Also, the crypts of the uterine mucous membrane lodge and retain the ovum either until it is impregnated, or until it dies, or is discharged.

If such views be accepted, it then becomes easy to understand the cause of tubal pregnancy. Desquamative salpingitis at once puts the tubes into a condition exactly similar to that of the uterus as regards its mucous membrane. In that condition retardation of the ovum in the tube will be inevitable, and access of the spermatozoa would be possible. The immediate adhesion of the ovum to the wall of the tube after impregnation would be as easy and as likely as its occurrence in the uterus itself.

Now, if the history of all these cases is taken, it is found that in nearly all of them there is prolonged sterility and menstrual suffering, showing that their procreative machinery was out of order. After the last confinement there has been an illness with marked symptoms of pelvic trouble, then many years of sterility, and then the ectopic gestation, ending in rupture.

The pregnancy having taken place in the tube, rupture must inevitably occur, and this will happen about the twelfth week, and it may occur as early as the fourth week, but the larger number of cases run on to about the twelfth. This tubal rupture takes two directions—(1) into the peritoneum; (2) into the broad ligament.

The symptoms of the first can hardly be mistaken. The sudden attack of pain, combined with all the signs of profound hæmorrhage, make the diagnosis only too certain, and unless an immediate operation is performed the woman will die in a very short period. The only treatment that can be proposed is to open the abdomen, and tie the bleeding vessels. The operation is comparatively easy. As soon as the peritoneum is opened the tube at fault is immediately detected and clamped if active hæmorrhage is still proceeding, and then rather a long process of washing out the abdominal cavity must ensue, and it must be continued until all the clots and *débris* are thoroughly removed, then a large glass drainage tube should be inserted, and the whole operation should not take more than from twenty to twenty-five minutes. In my last case, the patient, being only six weeks pregnant, was somewhat constipated and was straining at stool when rupture took place, and she was, picked up in a fainting condition. The abdomen was, up to the umbilicus, full of fluid and clotted blood. She was drained for fourteen days, and never really had a bad symptom after the operation. Speaking generally, it is apparently wiser to keep the drainage tube in the abdomen for at least ten days. The second variety of rupture into the broad ligament will not give such very characteristic symptoms. The hæmorrhage will not be so great, but if anything the pain is more pronounced, and is of a remittent type, occurring in a more pronounced way with each fresh hæmorrhage. On the death of the fœtus very frequently a miniature labour takes place, and a complete membranous cast of the uterus comes away. This is often so marked as to lead the medical attendant to think that he is dealing with an ordinary abortion; but if the pelvis is properly explored, it is soon discovered that the uterus is empty, and that the pelvic swelling is either behind or at the sides of the uterus.

It is none the less necessary to operate as soon as possible on these cases; on getting into the abdomen the sac must be torn through, the contents removed, and if possible the sac also, but if the adhesions are very firm it is much wiser to simply drain the sac and leave it to shrivel up, rather than run the risk of wounding the bowel or some big vessel to which the sac may be adherent. Any other form of treatment for these cases than abdominal section will only recommend itself to those who have only bad records to show in abdominal section.

It is fairly certain that all these cases must primarily occur in the Fallopian tubes, for it cannot be supposed that a fertilised ovum could drop into the peritoneal cavity and there become developed, for the powers of digestion of the peritoneum are so wonderful that it would have no chance of development.

